# Complete Genome Sequences of Eleven Cluster DJ *Gordonia rubripertincta* Bacteriophages

**DOI:** 10.17912/micropub.biology.001910

**Published:** 2026-01-16

**Authors:** Ping An, Meredith Barbieri, Dawn C. Bisi, Kristen Butela, Aimee H. Danley, Christina Grogan, Ben Hilldorfer, Bhaswati Manish, Kevin K. McDade, Eliza J. Neumann, Aparna Nigam, Rachelle Patterson, Ananya Saini, Samantha Walker, Otto G. Williams, Marcie H. Warner

**Affiliations:** 1 Department of Biological Sciences, University of Pittsburgh, Pittsburgh, Pennsylvania, United States; 2 Department of Math and Natural Sciences, Metropolitan Community College, Omaha, Nebraska, United States

## Abstract

Eleven novel cluster DJ *Gordonia rubripertincta* phages were isolated from soil collected in Pittsburgh, PA and Omaha, NE. The genomes of these phages have an average length of 60,185 bp and contain an average of 91 predicted genes. Genes with putative roles in structure, assembly, lysis, and nucleic acid processing were identified. Cluster DJ phages have an unusually low average G+C content (51.6%) compared to *G. rubripertincta* (~67%). These phages are also unusual in the organization of genes involved in lysis, the inclusion of two related major tail protein genes, and the absence of a candidate tail assembly chaperone gene.

**Figure 1. The morphological and genomic characteristics of 11 novel cluster DJ phages:**
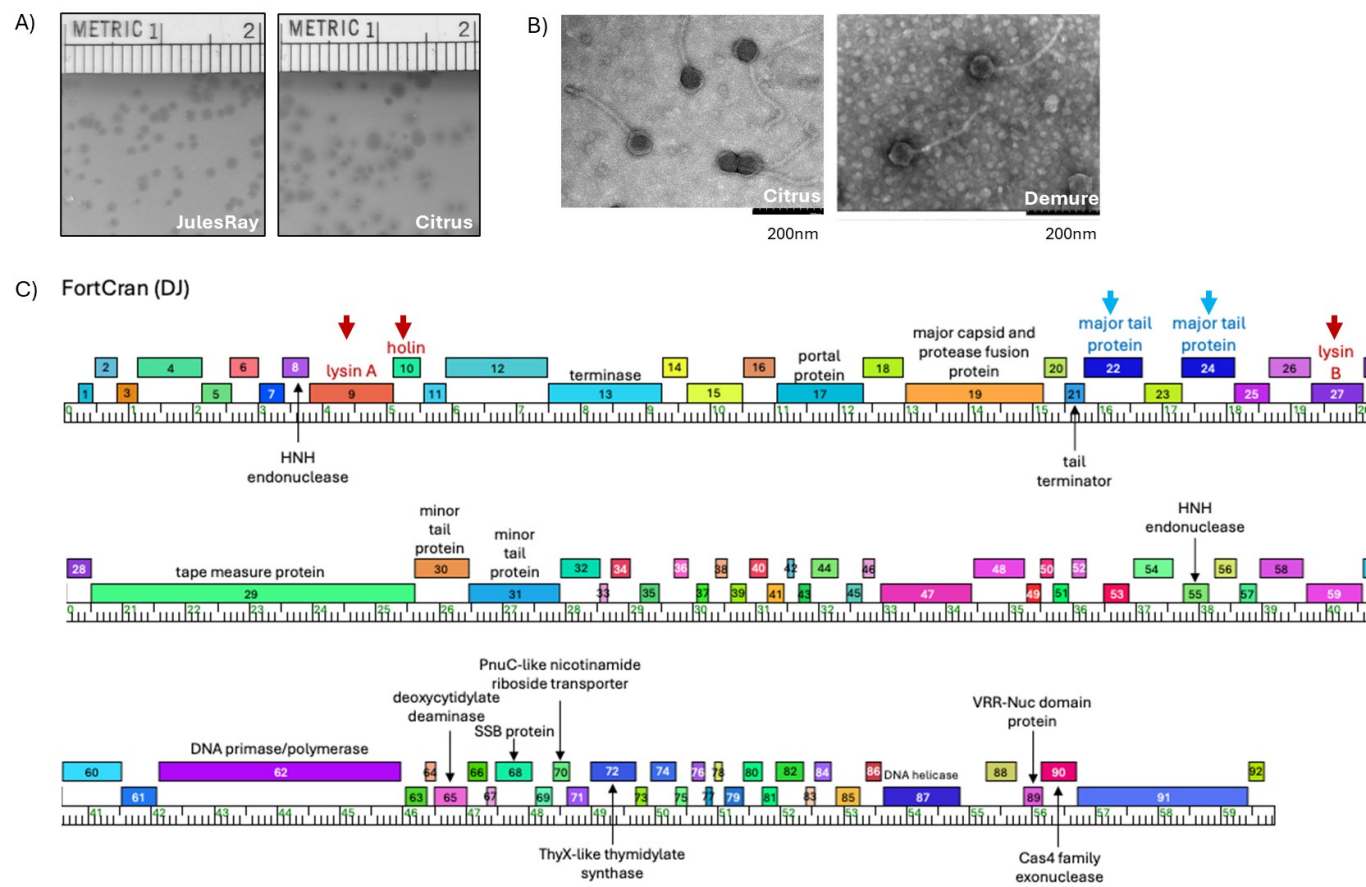
(A) DJ phages in this group tend to form clear plaques represented by the plaque morphologies of phages JulesRay (Left) and Citrus (Right). A culture of host cell *Gordonia rubripertincta* was inoculated with samples of JulesRay and Citrus separately. The phage and cell mixture were then combined with 0.4% PYCa top agar and overlaid on top of PYCa agar. Plates were incubated at 30 ^o^ C for 3 days. Sizes of the plaques range from 0.5 to 2mm in diameter. Numbers indicated by the rulers are in cm. (B) Morphology of viral particles was visualized using a HITACHI 7800 Transmission Electron Microscope following negative staining using 1% wt/vol uranyl acetate. Two representative pictures from phages Citrus (Left) and Demure (Right) are shown. A scale bar of 200nm length was included at the bottom right of each picture. (C) Several distinct genome features of DJ cluster phages were annotated using the Phamerator map of phage FortCran as a representative. Colored boxes represent protein coding genes in the genome. Numbers inside the boxes indicate gene numbers. Putative functions are listed above or below the corresponding gene boxes. The measuring tape indicates nucleotide coordinates and genes transcribed in the forward direction are positioned above the measuring tape. Genes involved in lysis were indicated using dark red arrows and text labels and the two major tail protein genes were indicated with blue arrows and labels. Additionally, note the lack of tail assembly chaperone gene and tRNA genes in the genome.

## Description

Due to advances in genome sequencing technology, the number of bacteriophage genomes that have been completely sequenced has increased rapidly in recent years (Hatfull, 2020, 2021; Heller et al., 2024; Pope et al., 2020).&nbsp; Despite this expeditious pace of identification and sequencing, novel phages are still frequently predicted to contain genes with no recognizable homology to those in previously identified phages; this suggests that phages still represent a substantial source of unexplored biological functions. We have focused our efforts on the identification of phages that infect the bacterial host *Gordonia rubripertincta* . This bacterium has potential applications in removal of pollutants from ecosystems (Frantsuzova et al., 2023); an understanding of the abundance and diversity of phages capable of infecting this organism may assist with assessing its viability as a remediation agent in natural environments. Furthermore, the genus *Gordonia* contains several opportunistic human pathogens (Barthel et al., 2023; Lesens et al., 2000; Vergov et al., 2023; Zheng et al., 2024) for which phage therapy may be a suitable treatment strategy.

This report focuses on the discovery and characterization of eleven novel cluster DJ *Gordonia rubripertincta* phages: Arataki, BetterYeti, Captrips, Citrus, Dancer, Demure, FortCran, Gusicorn, JulesRay, NoPickles, and ShaggyRogers. These phages were isolated from soil samples collected in Pittsburgh, Pennsylvania and Omaha, Nebraska during 2023 and 2024 (Table 1). Soil samples were first resuspended in phage buffer (10mM Tris-HCl, pH7.5, 68mM NaCl, 10mM MgSO _4_ , 1mM CaCl _2_ , 10% Glycerol) and agitated at 250 rpm at 30°C for 15 minutes to facilitate separation of phage particles from soil particulates. Samples were then centrifuged, and the supernatant was passed through a 0.22 μm filter to remove residual soil particulates and bacterial cells. For phages identified via direct isolation (Arataki, BetterYeti, Demure, NoPickles, and ShaggyRogers), the resulting filtrate was mixed with PYCa top agar (1.5% Peptone, 0.1% Yeast extract, 4.5mM CaCl _2_ , 0.1% Dextrose, 0.4% agar) containing *G. rubripertincta* NRRL B-16540 and overlaid on a solid PYCa agar (1.5% Peptone, 0.1% Yeast extract, 4.5mM CaCl _2_ , 0.1% Dextrose, 1.5% agar) plate. The resulting plates were incubated at 30°C for 3 days to allow for plaque formation. For phages identified utilizing an enrichment method (Captrips, Citrus, Dancer, FortCran, Gusicorn, and JulesRay), *G. rubripertincta* NRRL B-16540 and liquid PYCa were added to the soil filtrates, and the mixture was incubated at 30°C for 3 days with shaking to allow amplification of any *G. rubripertincta* phage in the sample. The cultured mixture was refiltered, and the supernatant was plated on a lawn of *G. rubripertincta* host cells embedded in PYCa top agar overlaid on a solid PYCa plate. Resulting plates were incubated at 30°C for 3 days to assay for host cell lysis due to phage activity. High titer phage lysates were prepared following two to three rounds of plaque purification. Phages in this group produced clear plaques, indictive of a lytic infectious cycle, that are frequently surrounded by haloes ( Figure 1A ). Transmission electron micrographs were obtained using a HITACHI 7800 Transmission Electron Microscope following staining with 1% uranyl acetate for 8 of the phages described in this report. All electron micrographs for this set of phages exhibit an icosahedral capsid ranging from 60 to 70nm in diameter and a flexible tail ranging from 235 to 260nm in length consistent with the morphology of siphoviruses (n=1 for each of the imaged phages, Figure 1B ).

To obtain viral genomic information, viral DNA was isolated from high-titer phage lysates using the Promega Wizard DNA Cleanup Kit. Individual libraries were constructed using the NEBNext Ultra II FS Kit and an Illumina MiSeq sequencer (v3 reagents) was used for sequencing. The approximate shotgun coverages of single-end 100 or 150 base reads varied between 955 to 15,696-fold (Table 1). For each viral genome, raw reads were assembled using Newbler v2.9 with default parameters (Russell, 2017). Genomes were manually checked for completeness using Consed v29 (Gordon & Green, 2013). The resulting genomes had an average length of 60,185 bp ranging from 59,584 to 61,136 bp, with an average G+C content of 51.6% (range 51.3% to 52%) (Table 1). All 11 phage genomes terminate in 9 base (CGCCGCTCT) 3' single stranded overhangs.

Initial auto-annotation was performed using GLIMMER v3.02 (Delcher et al., 1999) and GeneMark v2.5 (Besemer & Borodovsky, 2005) and manually refined using Phamerator v580 (Cresawn et al., 2011), DNA Master v5.23.6 (cobamide2.bio.pitt.edu), Starterator v587 (github.com/SEA-PHAGES/starterator), and PECAAN (discover.kbrinsgd.org). Functional assignments were evaluated using BLASTp v 2.2.26 (using NCBI nonredundant database and the database at phagesdb.org) (Altschul et al., 1990) and HHpred (using PDB_mmCIF70, PfamA, and NCBI Conserved Domain databases) (Soding et al., 2005). The presence of putative tRNAs was evaluated using Aragorn v1.2.38 (Laslett & Canback, 2004) and tRNAscan-SE v2.0 (Lowe & Chan, 2016). DeepTMHMM v1.0.42 (Hallgren et al., 2022) was utilized to evaluate the presence of predicted transmembrane domains in putative gene products. Default parameters were utilized for all programs other than DNA Master which was set according to instructions in the SEA-PHAGES bioinformatics guide (Pope, Jacobs-Sera et al., 2017).

All predicted genes in the eleven novel cluster DJ phages described in this report are transcribed unidirectionally. The finalized annotations of these genomes include between 90 and 93 protein coding genes (Table 1). Additionally, between 28 to 33 functional assignments were made in each genome (Table 1). Proteins with predicted roles in structure and assembly are interspersed throughout the 5’ end of the genome. The 3’ half of the genome contains many predicted genes that can be assigned putative functions in nucleic acid modification, processing, and metabolism. None of the genomes described in this report contain any predicted tRNA genes or genes associated with lysogeny.

Among actinobacteriophages, cluster DJ phages have previously been reported to have an unusual arrangement of lysis factors. The predicted lysin A and holin genes in these phages are unusually skewed toward the 5’ end of the genome at a position upstream of the predicted structural protein genes (Pollenz et al., 2022). Furthermore, while the genes encoding lysin A, holin, and lysin B are often found as a cassette of adjacent genes in actinobacteriophages (Pollenz et al., 2022), the gene that encodes the predicted lysin B in cluster DJ phages is found approximately 15 kb and 16 intervening genes downstream of the genes predicted to encode lysin A and holin ( Figure 1C ). All eleven phages reported here display this unusual arrangement of lysis factors.

Another atypical feature of cluster DJ phage genomes is the identification of more than one putative major tail protein gene ( Figure 1C ) (Benson et al., 2025). All 56 currently sequenced cluster DJ phages contain two major tail protein genes (FortCran genes 22 and 24) separated by a single gene for which a function has not been identified (FortCran gene 23). To explore the similarities and differences between the FortCran major tail protein genes, we conducted several comparative analyses. PhaMMseqs-grouping (Gauthier et al., 2022) of proteins with at least 35% amino acid identity and 80% sequence coverage, assigns both FortCran major tail proteins to the same “phamily” suggesting they likely share an evolutionary origin. We more closely examined the sequence variation between these genes using BLASTp; this analysis revealed that the FortCran major tail proteins share 43% amino acid identity (60% similarity) and that gene *22* encodes an additional 51 C-terminal amino acids. Further analysis demonstrates that the G+C content of gene 24 (49%) closely reflects the average G+C content of the eleven cluster DJ phages reported here (average 51.6%) while gene 22’s G+C content (63%) more closely aligns with the G+C content of *G. rubripertincta* (~67%) (Frantsuzova et al., 2025; Parveen et al., 2021). The sequence and G+C content variation between these genes suggests gene 22 was likely acquired as the result of horizontal transfer. It will be interesting to further explore the evolutionary histories of these genes and determine whether they perform overlapping or divergent functions.

Cluster DJ phages are also unusual in that, despite 56 manually annotated cluster members, the putative gene that encodes the tail assembly chaperones has not been identified ( Figure 1C ). Necessary for proper tail assembly, the tail assembly chaperone gene in actinobacteriophages is typically located immediately, or within a few genes, upstream of the gene encoding the tape measure protein (Christie et al., 2002; Levin et al., 1993; Xu et al., 2004). Interestingly, the only predicted gene in FortCran that aligns to a putative tail assembly chaperone gene is the single gene between the two major tail protein genes (gene 23 of FortCran). However, the homology and alignment are too incomplete for a functional designation and the gene lacks the characteristic slippery sequence that distinguishes this family of genes (Baranov et al., 2006; Christie et al., 2002; Levin et al., 1993; Xu et al., 2004). All cluster DJ members contain a predicted gene in this same gene family in the same position. Additional analyses will be required to determine if this protein has tail assembly chaperone activity, the tail assembly chaperones are located in an atypical genomic location, or if tail assembly occurs by an unexplored mechanism in the cluster DJ phages (Vladimirov et al., 2022).

**Table:** 

**Phage**	**Sample location (city, state, GPS)**	**Approx. read coverage (fold, x)**	**# of single end reads (size in bp)**	**Genome length (bp)**	**%GC content**	**# of ORFs (# with predicted function)**	**capsid diameter (nm)**	**tail length (nm)**
**Arataki**	Pittsburgh, PA 40.446795 N, 79.964165 W	15,696	10,605,554 (100)	60,488	51.60%	91 (31)	66	259
**BetterYeti**	Pittsburgh, PA 40.446884 N, 79.952998 W	3,659	2,690,994 (100)	60,438	51.90%	90 (30)	62	246
**Captrips**	Omaha, NE 41.171194 N, 96.032694 W	955	401,741 (150)	59,629	51.60%	91 (30)	ND	ND
**Citrus**	Pittsburgh, PA 40.442000 N, 79.956000 W	3,150	1,960,987 (100)	61,136	51.30%	90 (32)	68	240
**Dancer**	Pittsburgh, PA 40.260000 N, 79.570000 W	1,759	1,326,262 (100)	59,584	51.50%	92 (30)	ND	ND
**Demure**	Pittsburgh, PA 40.264600 N, 79.571100 W	4,305	2,724,930 (100)	60,622	51.60%	90 (28)	67	235
**FortCran**	Omaha, NE 41.223607 N, 95.990680 W	1,794	753,910 (150)	59,848	51.50%	92 (29)	70	260
**Gusicorn**	Pittsburgh, PA 40.440000 N, 79.960000 W	6,583	3,978,858 (100)	60,438	52.00%	92 (32)	60	240
**JulesRay**	Pittsburgh, PA 40.445983 N, 79.953041 W	3,844	3593453 (100)	59,943	51.60%	92 (32)	58	260
**NoPickles**	Pittsburgh, PA 40.443611 N, 79.955278 W	4,426	2,766,385 (100)	59,701	51.30%	93 (33)	ND	ND
**ShaggyRogers**	Pittsburgh, PA 40.443611 N, 79.955278 W	4,393	2,834,257 (100)	60,204	52.00%	91 (31)	66	257
**Average (Range)**				60,185 (59584-61136)	51.6 (51.3-52.0)	~91 (90-93) & ~31 (28-33)	(58-70)	(235-260)

**Nucleotide sequence accession numbers**

Arataki is available at GenBank with Accession No. PV915802 and Sequence Read Archive (SRA) No. SRX29486052 .&nbsp; BetterYeti is available at GenBank with Accession No. PV915818 and Sequence Read Archive (SRA) No. SRX29486053 . Captrips is available at GenBank with Accession No. PP978771 and Sequence Read Archive (SRA) No. SRX24892111 . Citrus is available at GenBank with Accession No. PV915797 and Sequence Read Archive (SRA) No. SRX29486054 . Dancer is available at GenBank with Accession No. PV915891 and Sequence Read Archive (SRA) No. SRX29486055 . Demure is available at GenBank with Sequence Read Archive (SRA) No. SRX29486056 (GenBank accession No. to be assigned). FortCran is available at GenBank with Accession No. PP978793 and Sequence Read Archive (SRA) No. SRX24892115 . Gusicorn is available at GenBank with Accession No. PV915881 and Sequence Read Archive (SRA) No. SRX29486057 . JulesRay is available at GenBank with Accession No. PV915826 and Sequence Read Archive (SRA) No. SRX29486058 . NoPickles is available at GenBank with Accession No. PV915879 and Sequence Read Archive (SRA) No. SRX29486052 . ShaggyRogers is available at GenBank with Accession No. PV915809 and Sequence Read Archive (SRA) No. SRX29486060 .
